# Intense remodeling of extracellular matrix within the varicose vein: the role of gelatinases and vascular endothelial growth factor

**DOI:** 10.1007/s11845-020-02289-1

**Published:** 2020-06-27

**Authors:** Anna Horecka, Anna Hordyjewska, Jadwiga Biernacka, Wojciech Dąbrowski, Tomasz Zubilewicz, Agnieszka Malec, Irena Musik, Jacek Kurzepa

**Affiliations:** 1 Department of Medical Chemistry, Medical University of Lublin, Chodźki 4A,, 20-093 Lublin, Poland; 2 I Clinic of Anaesthesiology and Intensive Therapy with Clinical Paediatric Department, Medical University of Lublin, Jaczewskiego 8, 20-090 Lublin, Poland; 3 Department of Vascular Surgery and Angiology, Medical University of Lublin, Staszica 17, 20-081 Lublin, Poland; 4 Department of Obstetrics and Pathology of Pregnancy, Staszica 16, 20-081 Lublin, Poland

**Keywords:** Gelatinases, Varicose veins, Vascular endothelial growth factor

## Abstract

**Background:**

Increased blood pressure in the varicose veins (VV) can contribute to the overexpression of matrix metalloproteinases (MMPs), affecting the endothelium, smooth muscle, and extracellular matrix of the vein wall. Gelatinases (MMP-2 and MMP-9), hypoxia, and inflammation occurring in the VV wall contribute to the increased expression of vascular endothelial growth factor (VEGF).

**Aims:**

Our objective was to analyze the concentration of gelatinases and VEGF in the great saphenous VV wall and plasma of patients.

**Methods:**

In total, 65 patients (2nd degree according to clinical state classification, etiology, anatomy, and pathophysiology—CEAP classification) aged 22 to 70 were enrolled. Control veins (*n* = 10) were collected from the patients who underwent coronary artery bypass graft surgery. Control plasma (*n* = 20) was obtained from healthy individuals. Gelatinases and VEGF levels were measured with the usage of ELISA method.

**Results:**

A significant increase in MMP-9 (11.2 vs. 9.98 ng/mg of protein) and VEGF (41.06 vs. 26 ng/g of protein) concentration in VV wall compared with control veins was observed. A positive correlation between VEGF versus MMP-2 (*p* = 0.03, *r* = 0.27) was found in the VV wall. However, no correlation was found between the concentration of VEGF and MMP-9 (*p* = 0.4, *r* = 0.11) in the VV wall. In addition, no statistical differences between MMP-9, MMP-2, and VEGF levels in plasma of VV patients compared with controls were noticed.

**Conclusions:**

The results of the present study confirm that VV’s patients have altered expression of MMPs and VEGF. Overexpression of MMP-9 and VEGF in the VV wall may contribute to the spreading of inflammatory process and suggests the intense remodeling of extracellular tissue within the VV wall.

## Introduction

Chronic venous insufficiency (CVI) is an important medical problem in developed countries. Increased blood pressure in the varicose veins (VV) can contribute to the overexpression of selected matrix metalloproteinases (MMPs), affecting the endothelium, smooth muscle, and extracellular matrix proteins of the vein wall [1, 2]. Gelatinases, which include MMP-2 (gelatinase A) and MMP-9 (gelatinase B), are responsible for the degradation of extracellular matrix (ECM) within the vein wall under both physiological and pathological conditions [3]. The main function of gelatinases involves degradation of fibers of denatured collagen but also the basement membrane and other structural components of ECM allowing migration of cells including smooth muscle cells [3, 4]. MMP-2 is constitutively secreted by smooth muscle cells and vascular endothelial cells [5]. MMP-9 is present in large quantities in the granules of neutrophils. It plays a major role in the influx of leukocytes to the site of infection or damaged tissue during inflammatory processes [2].

Vascular endothelial growth factor (VEGF) stimulates the synthesis of MMPs, especially MMP-9 [5, 6]. Hypoxia and inflammation occurring in the VV wall contribute to the increased expression of VEGF in the connective tissues [7]. VEGF plays an important role in maintaining the integrity of blood vessel walls and during the process of angiogenesis [7]. It interferes with the integrity of the vascular wall and cell homeostasis by increasing the endothelial permeability [7]. This results in swelling and the formation of “fibrin cuffs” characteristic of CVI. In addition, VEGF activates endothelial nitric oxide synthase (eNOS), which dilates venous vessels [7, 8]. Impaired synthesis of VEGF may be a predictor of vascular diseases.

As the mechanisms leading to the formation of the VV are still not fully understood, the objective of our study was to analyze the concentration of gelatinases and VEGF in the VV wall and in the plasma of patients with VV as the potential agents involved in VV pathogenesis.

## Methods

### Characteristics of the study group

Sixty-five patients (♀49, ♂16) aged 22 to 70 were enrolled (Table 1). The sample included patients with lower limb venous disease assigned as the 2nd degree according to clinical state classification, etiology, anatomy, and pathophysiology (CEAP), with varices present in the great saphenous vein [9]. Patients underwent VV surgery at the Institute of Rural Health in Lublin. The presence of thrombophlebitis or deep vein thrombosis, limb ischemia, clotting disorders, inflammatory diseases or cancer, diabetes mellitus, collagen diseases, surgical interventions in the last 12 months, and use of steroids and intravenous drugs in last 12 months excluded the patient from the study.

**Table 1:** Characteristics of the study group

	VV wall	Control 1	Control 2	*t* test
*n* (sex)	65 (♀49, ♂16)	10 (♀8, ♂2)	20 (♀11, ♂9)	NA
Age (years) Mean age ± SD (years)	22 to 70 60 ± 11.002*	50 to 75 62 ± 8.3	29 to 62 56 ± 10.2	*p* > 0.05*
BMI	22.4 ± 3.1*	23.1 ± 2.3	22.9 ± 2.9	
Hypertension (yes/no)	12/53	4/6	7/13	

*Difference between either VV wall group vs. control 1 or control 2

### Study material

The material of VV patients was collected from femoral segment of varicose great saphenous vein using Babcock method and patients’ blood was collected from the antecubital vein during surgery. Control groups consist of two subgroups. The first one includes control group no. 1 (*n* = 10, ♀8, ♂2, aged 50 to 75)—patients who underwent coronary artery bypass graft (CABG) surgery, with no symptoms of VV and no retrograde flow proven by duplex ultrasound (DU) whose great saphenous veins were removed. The second one is control group no. 2 (*n* = 20, ♀ 11, ♂ 9, aged 29 to 62)—healthy individuals whose blood was collected from the antecubital vein.

### Sample preparation

Blood sample (VV and control no. 2) was collected from the antecubital vein into tubes with lithium heparin as an anticoagulant. Then it was centrifuged at 3000 rpm to obtain plasma, in which gelatinases and VEGF concentration was determined. Next 0.5 g of venous walls (VV and control no. 1) was homogenized in 5 ml of buffer containing 0.1 M Tris–HCl pH 7.4 and centrifuged for 15 min at 3000 rpm. The prepared material was stored at temperature − 70 °C. Gelatinases and VEGF levels were measured in plasma and vein walls of VV patients and both control individuals (control no. 1 and control no. 2).

### Gelatinases and VEGF measurements

Commercially available diagnostic kits, Human MMP-2 Quantikine ELISA Kit and Human MMP-9 Quantikine Immunoassay (R&D System, Abingdon, UK), were applied to gelatin concentration evaluation. The gelatinase levels were expressed in nanograms per milliliter in plasma and in nanograms per milligram of protein in vein wall homogenates. VEGF concentration was determined using diagnostic kit Human VEGF Quantikine Immunoassay (R&D System, Abingdon, UK) and expressed in picograms per milliliter in plasma, and nanograms per gram and nanograms per milligram of protein in tissue. Protein level in tissue samples was estimated with the usage of commercially available Bradford reagent (BIO-RAD Protein Assay, Hercules, USA). The assays were performed with the usage of Thermoshaker DTS-4 (ELMI, Calabasas, North America) and Microplate Reader Model 680 (BIO-RAD, Hercules, USA) with software Microplate Manager version 5.2.1 (BIO-RAD, Hercules, USA). All measurements were performed according to manufacturers’ manuals.

### Statistical analysis

*T* test was applied in case of parametric distribution of values whereas the difference between values with non-parametric distribution was checked with Mann–Whitney *U* test. Verification of hypothesis was performed at the significance level *α* = 5% (*p* < 0.05). Values are expressed as mean ± SD (Gaussian distribution) or median and 1st–3rd quartiles (non-Gaussian distribution). Spearman correlation coefficient has been used to establish the relationship between MMP-9, MMP-2, and VEGF concentration in VV wall. Statistical analysis was done with GraphPad InStat v. 3.10 (San Diego, USA).

## Results

**Fig. 1:**
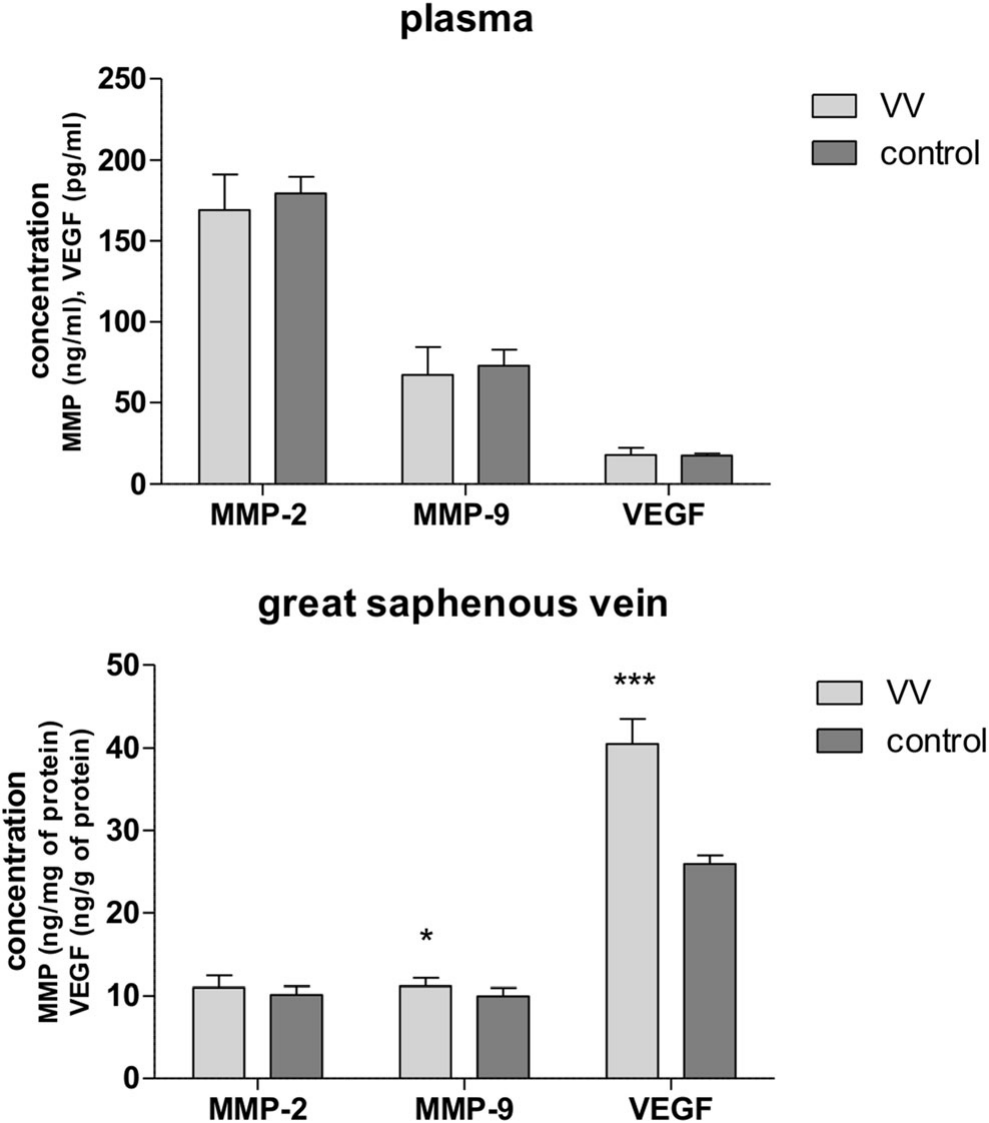
Changes in concentration of gelatinases and VEGF in plasma and great saphenous veins of VV patients. Data are means (SD) or median (1st–3rd quartile); ***p < 0.001, Mann–Whitney *U* test; *p < 0.05, *t* test

**Fig. 2:**
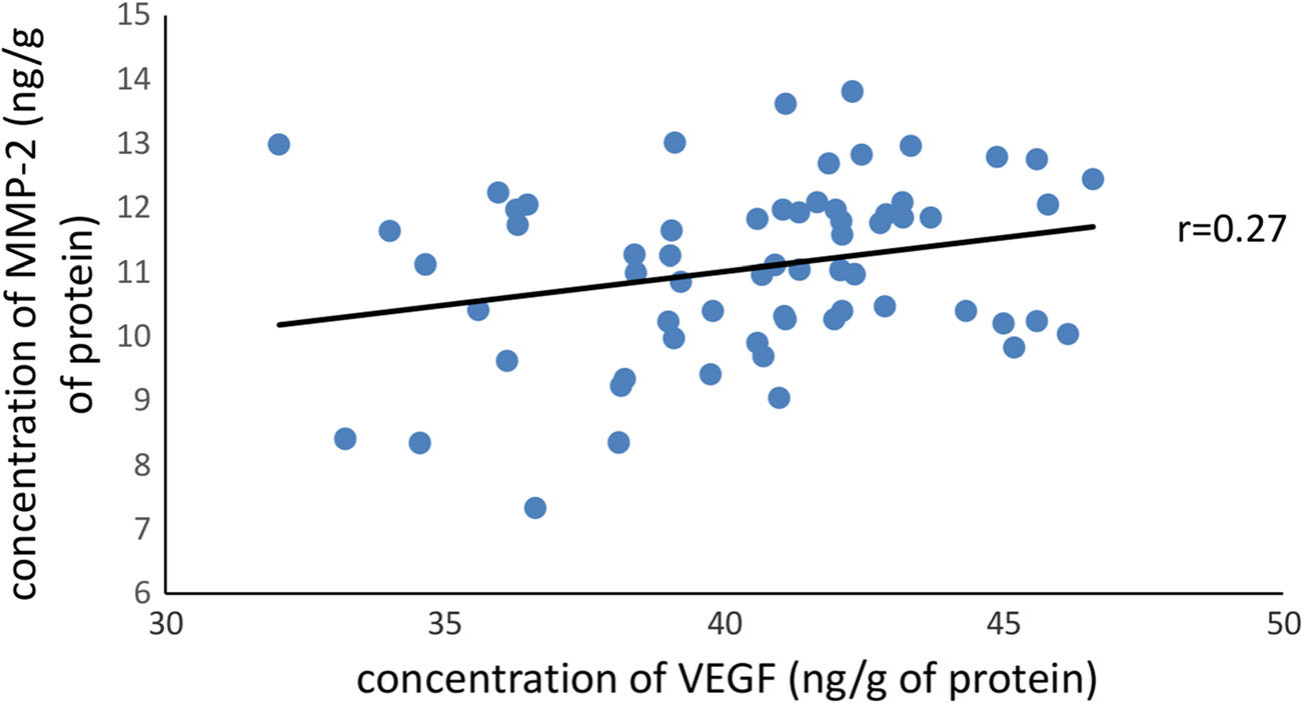
A positive correlation between the concentration of VEGF vs. MMP-2 in the VV wall (Spearman correlation coefficient *p* = 0.03, *r* = 0.27)

A significant increase in MMP-9 (11.2 vs. 9.98 ng/mg of protein) and VEGF (41.06 vs. 26 ng/g of protein) concentration in VV wall compared with control no. 1 was observed (Fig. 1). No statistical differences between MMP-9, MMP-2, and VEGF levels in plasma of VV patients were noticed compared with controls. A positive but weak correlation between the concentration of VEGF versus MMP-2 (*p* = 0.03, *r* = 0.27) was found in the VV wall (Fig. 2). However, no correlation was found between the concentration of VEGF and MMP-9 (*p* = 0.4, *r* = 0.11) in the VV wall.

## Discussion

Changes in the activity of MMPs and VEGF were observed in many diseases of the circulatory system [10–13]. The VV development is associated with reduced wall thickness, changes in hemodynamics, the flow of inflammatory cytokines, changes in the ECM, and increased generation of reactive oxygen species (ROS) affecting the activity of MMPs [14–17]. Damaged epithelium causes the recruitment and adhesion of leukocytes, which play a significant role in the inflammatory process and lead to the damage of vessel walls. Prolonged oxidative stress in the VV wall is associated with the increased expression of gelatinases and hypoxia-inducible transcription factors (HIF): HIF-1α responsible for the transcription of approximately 150 different genes including VEGF [5]. A positive MMP/TIMP ratio can cause excessive degradation of ECM leading to structural changes in the vein wall including valvular dysfunction that is one of the major factors affecting the development of VV [15, 18]. It was demonstrated that the patients with VV have the increased activity of several MMPs (− 1, − 3, − 13) in plasma and tissues [19, 20]. The changes in the content of collagen in the VV wall, especially in the VV complicated by thrombophlebitis, can be caused by increased gelatinase activity [21, 22].

The previous results related to gelatinase activity in VV were ambiguous. The activity of MMP-2 was found to be decreased [23] or not changed [24–27] in VV wall in comparison with normal tissue. The increased activity of MMP-2 has been also found in the model of VV’s cell line culture [28]. Our study revealed no statistically significant fluctuation of MMP-2 in the VV wall and plasma of patients. Its constitutive expression causes the MMP-2 level to be unsusceptible for pro-inflammatory cytokine fluctuation.

MMP-9 is important for maintaining the proper tension of blood vessel wall [2]. Serra et al. noticed the elevated level of MMP-9 and neutrophil gelatinase-associated lipocalin (NGAL) in the plasma of patients with venous ulcers [29]. Other reports demonstrated no changes [26, 30] in activity of MMP-9 or even decreased activity [31] in the VV. It was postulated that MMP-9 can be involved in the degradation of the vessel wall mainly of the media layer of vascular smooth muscle [29], whereas Huh et al. observed the increased expression of MMP-9 in endothelial cells of vascular smooth muscle [27]. In our study, a significant increase in MMP-9 concentration within VV wall compared with control no. 1 was observed. Higher MMP-9 level can be caused by elevated VEGF level, which is one of the most important inducers of MMP-9 expression. Overexpression of MMP-9 in the VV wall indicates the presence of the inflammatory process. This result supports the previous observations that MMP-9 can contribute to VV development [27]. The differences between MMP-9 level in study and control plasma were not statistically significant.

VEGF is considered to be the most potent stimulator of angiogenesis [32]. In addition, VEGF activates endothelial nitric oxide synthase (eNOS), leading to the increase of venous hypertension and blood stasis [8]. VEGF increases the permeability of existing blood vessels, helping to maintain the inflammation, by enabling the migration of leukocytes to the final destination. It proved to be 50,000 times more active than histamine [7]. Besides, it participates in the reconstruction of the ECM. Kowalewski et al. evaluated the content of VEGF-A and its receptors (VEGF-R1, VEGF-R2) in the VV wall and VV wall complicated by thrombophlebitis. It was found that in the VV wall, expression of VEGF-A and VEGF R2 is increased. Expression of VEGF-A and VEGF R1 in the VV wall complicated by thrombophlebitis is increased compared with the VV wall and control tissue [8]. Flórez et al. confirmed the increased expression of VEGF in the VV wall [12]. Based on these results, it can be concluded that VEGF is overexpressed in the VV wall. In subsequent years, it was found that the poor performance of the saphenous vein is associated with changes in gene expression of VEGF (VEGF121/VEGF165) and its receptor (KDR, flt-1, s.flt-1). The authors suggested that changes in the transcription of VEGF121 and s.flt-1 SFJ can be used as predictors in the early stage of the VV [33]. Our study confirmed the results of the previous researches. We observed increased levels of VEGF in VV vein probably due to the fact that the agent is induced in CVI patients in response to tissue injury caused by venous hypertension. In the case of plasma, the result is not statistically significant.

However, to the best of our knowledge, this is the first study evaluating the correlation between the concentration of gelatinases and VEGF in the VV wall. Wojcik et al. showed a significant correlation between the level of VEGF and MMP-9 in plasma of patients with small cell lung cancer that is associated with increased angiogenesis [34]. Both gelatinases are involved in cancer metastasis [35]. A similar correlation was found in the fluid collected from the vitreous body of diabetic retinopathy patients [36]. In the case of gastric cancer, a positive correlation between the concentration of the VEGF and MMP-2 and also VEGF and MMP-9 was found [34]. Our study showed positive correlation between VEGF and MMP-2 concentration in the VV wall. MMP-2 is a constitutive enzyme and VEGF directly affects its activation [19, 34, 37].

In conclusion, the results of the present study confirm that VV patients have altered expression of MMP-9 and VEGF. Overexpression of MMP-9 and VEGF in the VV wall may contribute to the spreading of inflammatory process and suggests the intense remodeling of extracellular tissue within the VV wall. The conducted study shows the coexistence of VV with elevated concentrations of VEGF and MMP-9 in the VV wall. The research does not determine whether it was the primary cause of VV or the increase in MMP-9 and VEGF.
